# Complete Genome Sequences of *Bacillus* Phages DirtyBetty and Kida

**DOI:** 10.1128/genomeA.01385-16

**Published:** 2017-03-09

**Authors:** Kelly Flounlacker, Rachel Miller, Diana Marquez, Allison Johnson

**Affiliations:** Center for the Study of Biological Complexity, Virginia Commonwealth University, Richmond, Virginia, USA

## Abstract

We report here the genome sequences of two bacteriophages of the *Bacillus cereus* group, DirtyBetty and Kida. These bacteriophages are double-stranded DNA-containing *Myoviridae* isolated from soil samples using *Bacillus thuringiensis* subsp. *kurstaki* as their host bacteria.

## GENOME ANNOUNCEMENT

The ATC family of the genus *Bacillus* includes the spore-forming soil bacteria *B. cereus*, *B. anthracis*, and *B. thuringiensis*. The bacteria in the ATC family are very closely related genomically but display pathogenic differences (1, 2). It is of interest to discover phages which can be isolated safely by students using *B. thuringiensis* to learn more about these phages and their host bacteria in the ATC group. Students in the VCU Phage Lab, as part of the Howard Hughes Medical Institute (HHMI) SEA-PHAGES program (3), have isolated bacteriophage infecting *Bacillus* spp. for the past 3 years. These phages are generally *Myoviridae* with isometric heads containing double-stranded DNA and long contractile tails.

Phages Kida and DirtyBetty were isolated from soil samples collected in Richmond and Vienna, VA, respectively, using enrichment with *B. thuringiensis* subsp. *kurstaki* as the host bacterium. Phages Kida and DirtyBetty both have head diameters of 100 nm, as well as tail lengths of 200 nm and 375 nm, respectively. Kida forms clear plaques on a lawn of *B. thuringiensis*, while DirtyBetty forms plaques with thin halos. The genomic DNA of these bacteriophages was sequenced by MiSeq next-generation sequencing technology to at least 45-fold coverage, assembled by the Newbler software, and visualized by Consed. The physical genome ends containing long terminal repeats were determined by identifying a region with approximately double read coverage. DNA Master (http://cobamide2.bio.pitt.edu/computer.htm) was used to annotate the genes in each genome, and the software tool integrates GeneMark (4) and Glimmer (5) for predicting open reading frames and Aragorn (6) to predict the presence of tRNA genes. Functional predictions were derived from BLASTP (7) conserved domains and HHpred (8) and by comparing each gene to homologs in the Phamerator (9) comparative genomics software.

The Kida genome has a length of 162,151 bp, with long terminal repeats at a length of 2,574 bp. DirtyBetty has a genome length of 162,415, with 2,808-bp long terminal repeats. Both genomes have 38.7% G+C content and have 300 (DirtyBetty) and 304 (Kida) predicted open reading frames. Neither phage has tRNAs present in its sequence. The two phages share 94% query coverage and 95% identity to each other, as well as similarly high values to other published cluster C1 *Bacillus* phages (10–13). They share 278 protein families (92% of their proteins), or phams (as described in reference 9), and relatively few protein-coding genes that are present in only one of these phages. Furthermore, only 15% of these proteins (44/300 for DirtyBetty and 43/304 for Kida) have a predicted function, generally for structural, lytic, and nucleotide metabolism genes. There are no genes in these genomes without at least one homolog in our *Bacillus* phage database (http://bacillus.phagesdb.org/), which indicates that no novel genes are present in these two phages. While the genes with predicted function are conserved in these phages and other closely related cluster C1 phages, this group also contains a large set of conserved proteins with no identifiable homologs outside this group, providing a rich data set for determination of gene essentiality and function.

### Accession number(s).

The complete genome sequences of the *Bacillus* phages DirtyBetty and Kida are available in GenBank under accession numbers KX349903 and KX349902, respectively.
